# Deixis, Meta-Perceptive Gaze Practices, and the Interactional Achievement of Joint Attention

**DOI:** 10.3389/fpsyg.2020.01779

**Published:** 2020-09-11

**Authors:** Anja Stukenbrock

**Affiliations:** Chair of German Linguistics, University of Lausanne, Lausanne, Switzerland

**Keywords:** reference, deixis, joint attention, gaze following, mutual monitoring, sequential organization, social interaction

## Abstract

The paper investigates the use of gaze along with deictics and embodied pointing to accomplish reference and joint attention in naturally occurring social interaction. It assumes that deixis, in its primordial use in face-to-face interaction, is an embodied phenomenon that involves gestural pointing as well as visual perception, thus giving rise to recurring gaze practices of the participants. The analysis draws on a model of the interactional organization of deictic reference and joint attention that serves as a sequential framework for investigating the functions of eye gaze. The analysis focuses on two meta-perceptive practices: gaze following and gaze monitoring. It shows that the use of these practices in naturally occurring social activities is context dependent, positionally sensitive, tied to participant roles, and temporally fine-tuned to the stream of the participants’ verbal and embodied conduct. The sequential analysis of these practices further documents that meta-perceptive gaze practices contribute to the constitution of joint attention as mutually known by the participants. The data for this study were recorded with two pairs of mobile eye tracking glasses and an external camera. Methodologically situated within the framework of conversation analysis and interactional linguistics where video recording is used, the study breaks new ground by employing a technology almost exclusively applied in experimental frameworks to record ordinary social activities “in the wild.” In striving for ecologically valid and precise eye gaze data, it also contributes to a refinement of concepts developed in experimental paradigms by adapting them to qualitative research within the field of multimodal conversation analysis and interactional linguistics.

## Introduction

Across languages, demonstratives, or deictics, constitute a particular class of linguistic items. They are defined by a range of features that distinguish them both from grammatical and lexical items: they are universal (Himmelmann, 1997; Diessel, 1999, 2006); they have developed so long ago that they cannot be reconstructed diachronically from either lexical or grammatical items (Diessel, 1999, 2006); in ontogenesis, they are among the first words that children acquire (Clark, 1978); they constitute the only linguistic class that is inextricably connected to gestures (Bühler, 1965 [1934]; Diessel, 2006; Stukenbrock, 2015); and they fulfill one of the most central functions in human communication: the establishment of joint attention (Diessel, 1999, 2006).

There are various ways in which we can direct a person’s attention to an external object. An important attention-directing device is gestural pointing, which precedes the acquisition of verbal deictics and is considered to be universal (Povinelli and Davis, 1994; for a different view cf. Wilkins, 2003) and uniquely human (Butterworth, 2003; for a differentiated overview of great apes’ capacities for imperative vs. declarative/referential pointing cf. Tomasello and Call, 2019). Whether established verbally and/or gesturally, joint visual attention constitutes a triadic relationship between two participants and an object. This implies that they shift their gaze to the object. In face-to-face interaction, these gaze shifts may be observed and interpreted by the participants with respect to the ongoing activities. Eye gaze thus assumes an interactional function (Rossano, 2012, 2013). It shapes participants’ actions and mutual understandings of those actions. Gaze may also serve to establish joint attention in the absence of verbal and/or gestural pointing. By following another person’s line of regard (Flom et al., 2007), and by observing and inferring what the other person sees, we may share attention with that person. Note that joint attention involves more than following another person’s gaze, or gesture, to an object. Joint attention presupposes two (or more) persons focusing on the same phenomenon and being aware of that act (Clark and Marshall, 1981; Moore and Dunham, 1995). In other words, joint attention must be mutually known in order to become part of the participants’ common ground (Clark, 1996).

How do participants in the course of demonstrative reference know that joint attention has occurred? How do they make sure that they are seeing the same phenomenon, and see it in the same way? How do they achieve a mutual understanding of the referent? And how does gaze help them shape their actions according to the actions of the other? The present paper is concerned with these questions. It investigates the use of deictics, pointing, and gaze to accomplish joint attention in naturally occurring social activities. It assumes that the use of deictics in face-to-face interaction is inexorably connected to embodied, visible acts of demonstration. It proposes that eye gaze constitutes an integrative component of how joint attention is cooperatively accomplished. Coordinating joint actions is less successful when participants have limited or no visual access to the same objects and/or to each other (Clark and Krych, 2004). In the process of jointly attending to objects, participants look at each other at key moments in order to design and time their actions with respect to the actions of the other.

This paper aims at identifying those moments by focusing on the gaze behavior that participants themselves orient to in the course of demonstrative reference. By checking the gaze orientation of their coparticipant, they attribute relevance to the other’s perception. The inferences that participants draw from perceiving the other’s perception are displayed subsequently in their own behavior. In turn, these displays of understanding shape the participants’ next actions and as such become accessible to sequential analysis (Schegloff, 2007). Gaze practices that turn perception into an object of perception will be termed meta-perceptive gaze practices:^1^ a person (*ego*) looks at the eyes of another person (*alter*), perceives the other’s gaze orientation as an index of visual perception, and interprets it within the context of its occurrence. Two types of gaze practices will be investigated: *gaze following* and *gaze monitoring*. While the former is tied to the participant role of the addressee who follows the speaker’s gaze, hence *speaker gaze following*, the latter is performed by the speaker to check the gaze orientation of the addressee, hence *addressee gaze monitoring*.

I begin by discussing previous research on deixis, gesture, and gaze. Subsequently, I introduce a model of deictic reference that provides the framework for analyzing the meta-perceptive gaze practices in the context of demonstrative reference and joint attention (section “A Model of the Interactional Organization of Deictic Reference and Joint Attention”). Next, a note on materials and methods explains how mobile eye tracking was applied within the framework of conversation analysis (section “Materials and Methods”). This is followed by sequential analyses of deictic reference and meta-perceptive gaze practices within everyday activities (section “Analysis: Meta-Perceptive Practices and Joint Attention in Deictic Reference”). The paper concludes with a discussion of the findings (section “Discussion”).

### Conceptualization of Deixis as an Embodied Phenomenon

In research on deixis, two theoretical traditions can be distinguished. In the Anglo-American tradition, the terms *deixis* and *indexicality* are used “coextensively” (Levinson, 2004, p. 97) and apply to the broader phenomenon of context dependency. In contrast, the European tradition within which my paper is situated favors a narrow definition. Following Bühler (1965 [1934]), the concept of *deixis* refers exclusively to the grammatical encoding of context dependency in a closed set of linguistic items (deictics/demonstratives). Deictics have grammaticalized the space-, time-, and person-bound structure of the participants’ subjective orientation (*origo*) in the speech event (Bühler, 1965 [1934]). Bühler distinguishes three modes of deictic reference: (1) *demonstratio ad oculos et ad aures* (reference to visible phenomena in the surroundings), (2) *anaphora* (reference to elements in the context of speech), and (3) *Deixis am Phantasma* (reference to absent phenomena that have to be imagined). Only *demonstratio ad oculos* is relevant for the present paper. The Latin syntagma that Bühler chose (*demonstratio* = “pointing” and *ad oculos* = “to/for the eyes”) links the speaker’s gesture to the addressee’s eyes, thus anticipating an understanding of deixis as an embodied, interpersonal phenomenon.

Bühler postulated that gestures constitute an indispensable component of verbal deixis (Bühler, 1965 [1934], p. 93). In the Anglo-American tradition, this was also acknowledged by Fillmore who distinguished gestural from symbolic and anaphoric usages: gesturally used deictics “can be properly interpreted only by someone who is monitoring some physical aspect of the communication situation” (Fillmore, 1997 [1971], p. 62), notably, the speaker’s gesture, body orientation, gaze direction, etc. Various strands of video-based research have since contributed to an understanding of deixis and gesture in spoken interaction. They provide a point of departure for including gaze in the discussion.

In gesture studies, Kendon (1988, 2004) laid out a continuum of gesture types, later termed “Kendon’s continuum” (McNeill, 1992, McNeill, 2000): different types of pointing gestures were systematically described (Kendon and Versante, 2003; Kita, 2003; Kendon, 2004), including the use of different body parts such as lips (Sherzer, 1973; Enfield, 2001), nose (Cooperrider and Núñez, 2012), and eye gaze (Kendon, 1967; Streeck, 1988, 1993, 2002; Stukenbrock, 2015). Conversation analytic studies have sharpened our understanding of demonstrative reference as an interactional achievement that requires coordinating talk, gestures, gaze, and body movements (Streeck, 1988, 1993, 2002; Goodwin, 2000, 2003; Hindmarsh and Heath, 2000; Hausendorf, 2003; Mondada, 2005, 2007, 2012, 2014; Stukenbrock, 2008, 2009, 2010, 2015; Eriksson, 2009). These works show that the use of deictics and concurrent gestures form multimodal packages, or *Gestalts*, that are recipient designed (Sacks et al., 1974; Hindmarsh and Heath, 2000), and coupled with the environment in which they occur (Goodwin, 2003, 2007). An integrative account of demonstratives and gestures as closely coupled, temporally flexible resources includes describing the interaction between speaker and addressee gaze.

### Multimodal Deixis and Gaze: Temporality, Interactivity, and Intersubjectivity in Context

In face-to-face interaction, gesturally used deictics demand that addressees shift their gaze away from the speaker and to the target object. They constitute a summons that makes a response of the addressee relevant, the response being visual attention by means of gaze allocation. The idea that features in the speaker’s talk instantiate a summons for addressee gaze was first formulated by Goodwin (1980, 1981): restarts, pauses, and hesitations produced by the speaker request the gaze of a non-gazing hearer (Goodwin, 1981, p. 280). A similar relationship holds for the use of deictics and addressee gaze, with an important difference: the gaze summons implemented by a demonstrative signals that the addressee is expected to look at the speaker’s body to gather visual information on the location of the object (Stukenbrock, 2018c). In short, whereas restarts, pauses, and hesitations summon the addressee to establish mutual gaze and a *dyadic relationship* with the speaker, deictics summon the addressee to participate in a *triadic relationship*, i.e., to look at the speaker and pick up embodied cues to locate the object.

Although conversation analytic studies have revealed the multimodal complexity of deixis and have shed some light on gaze (Hindmarsh and Heath, 2000; Goodwin, 2003; Eriksson, 2009; Stukenbrock, 2009, 2015; Mondada, 2014), to date, they rely exclusively on video recordings that do not allow to zoom in on the details of gaze. In contrast to video recordings, mobile eye tracking technology delivers information on the location and duration of target fixations, the trajectories of gaze shifts, and, last but not least, on the interaction between speaker and addressee gaze.

While eye trackers have been used in experimental studies to examine joint attention and reference (Louwerse and Bangerter, 2005, 2010; Land, 2006; Hanna and Brennan, 2007; Clark and Gergle, 2010; Gergle and Clark, 2011), mobile eye tracking studies on deictic reference in naturally occurring interaction are practically non-existent (see, however, Stukenbrock, 2018a,b; Stukenbrock and Dao, 2019). In contrast, experimental studies on gaze and reference are undertaken in highly controlled settings, most of them stationary with the participants seated. For instance, the question how participants’ gaze patterns interrelate was tackled in dual eye tracking experiments on the coordination of gaze in a programming task in which two programmers worked on the same code on two different computers placed opposite one another. The concept of “gaze cross-recurrence” (Jermann and Nüssli, 2012) was introduced to capture how much participants looked at the same spots simultaneously, and a method was developed for the automatic extraction of gaze cross-recurrence in large amounts of experimental data.

In everyday life, however, reference often involves participants on the move as well as moving objects. Mobile configurations are both shaped by and shaping the participants’ use of verbal and visual resources (De Stefani, 2010; Haddington et al., 2013; De Stefani and Gazin, 2014; De Stefani and Mondada, 2014). Their investigation cannot be treated separately from the dynamically changing context that they help constitute. A few experimental studies have applied mobile eye tracking to compare reference in stationary and mobile settings. In a conversation elicitation task (Clark and Gergle, 2010; Gergle and Clark, 2011), participants in seated and standing mobile conditions were asked to discuss LEGO objects according to their likelihood of being replicas of modern art. The results showed differences between mobile and seated participants, i.e., mobile pairs used a higher proportion of local deictics for reference than seated participants but showed a lower proportion of gaze overlap (Gergle and Clark, 2011, p. 442). These results point to the context dependency and flexibility of participants’ solutions to the problem of establishing joint attention and thus underline the need to study them in the social contexts in which they are embedded.

This also holds for gaze following (Flom et al., 2007). It has been noted that gaze following includes “an inference about perception: The observer follows to see what the gazer perceives” (Brooks and Meltzoff, 2014, p. 171). In ordinary activities, these inferences are based on context- and activity-related attributions of intentionality and meaning. In experimental settings, however, gaze following is examined as a mechanism that involves an observer following directional gaze shifts of an experimenter in a decontextualized way. Its investigation plays a prominent role in developmental studies on the age relatedness of infants’ capacity for joint attention and for cooperative behavior (Scaife and Bruner, 1975; Carpenter et al., 1998; Flom et al., 2007; Tomasello et al., 2007), on the relationship between gaze following and language development (Brooks and Meltzoff, 2008), and on infants’ abilities to use first-person experiences to understand the visual experiences and minds of others (Brooks and Meltzoff, 2014). From an interactional perspective, gaze following in experiments is initiated by the experimenter who first establishes (eye) contact with the gaze follower and then shifts gaze to the object. However, in everyday life, gaze following emerges in particular spatial, temporal, and social contexts and assumes interactional significance within those contexts. Alternative paths such as eye–hand coordination between child-eye and adult-hand instead of eye gaze following may be preferred by social partners in more natural, free play contexts with several targets (Yu and Smith, 2013). Note that, in the flow of everyday activities, gaze following may just be an evanescent event that does not become relevant in interaction. Without uptake, however, participants may not be aware of its occurrence. This, then, does not constitute joint attention as mutually known by the participants (Clark, 1996).

In sum, experimental studies face three problems that affect the transferability of their results to human interaction “in the wild:” the problem of temporality, the problem of interactivity, and the problem of intersubjectivity. Depending on the interactional, cognitive, and perceptual availability of the participants, the interactional accomplishment of joint attention follows different temporal orders. Participants establish joint attention not only *simultaneously* but also *successively*, i.e., when speakers withdraw their gaze from the object before addressees look at it. Whereas instances of the first case lead to “gaze cross recurrence” (Jermann and Nüssli, 2012) in the eye tracking data, instances of the second case do not. Accomplishing joint attention implicates more complex, reciprocally adaptive gaze patterns of the participants than looking at the same target. These include mutual gaze as well as meta-perceptive practices such as gaze following and gaze monitoring. For instance, a common practice that infants have yet to acquire (Franco and Butterworth, 1996; Liszkowski et al., 2004) involves a pointing speaker (P) checking the visual orientation of the addressee (A) at the moment when A is looking at the target. In the eye tracking data, this appears as a fixation of P’s gaze cursor on A’s eyes at the moment in which A’s gaze cursor reveals a fixation on the target. This moment of P perceiving A’s perception constitutes an interactional mechanism for P to make inferences about A’s perception and understanding.

Given that the eye tracking data reveal that the gaze of P and A is on the same target, the participants themselves may not be aware of the fact that they are looking at the same thing. Even if they know that they do, they may (or may not) find out subsequently that they have constructed different referents. As targets and referents are not the same, they need to be distinguished (Quine, 1960, p. 29ff; Clark et al., 1983, p. 245ff; Stukenbrock, 2015, p. 72–85, 282–313) to avoid naive conclusions about reference solely on the grounds of gaze fixations in the eye tracking data. Even though these fixations are technically precise, they do not reveal *prima facie* what the participants “really” see and what they referentially construct from what they see (Goodwin, 1994).

To resume, the problem consists of how to ground claims that (1) joint attention has occurred in the course of demonstrative reference, (2) mutual knowledge about the occurrence of joint attention exists, and (3) an intersubjective understanding of the referent has been achieved. I argue that participants themselves are continuously confronted with these problems and have developed routine solutions for them in social interaction. These solutions constitute the sequential orderliness of demonstrative reference in naturally occurring interaction. They are temporally flexible, context-sensitive, and thus serve participants’ practical needs in everyday life. The following model reconstructs those solutions as interactional jobs that participants fulfill to establish reference and joint attention.

## A Model of the Interactional Organization of Deictic Reference and Joint Attention

In this section, I present a model that conceptualizes deictic reference as an interactional accomplishment (Stukenbrock, 2015, p. 495) and accounts for the multimodality, temporality, and intersubjectivity of demonstrative reference in face-to-face interaction. The model specifies the interactional jobs that participants have to fulfill depending on their roles as referring/pointing speaker and addressee. The jobs are conceived of as sedimented solutions to participants’ concrete problems of sharing attention on visible phenomena in everyday life. While their jobs are complementary, both participants actively contribute to joint attention. The model was developed empirically on the basis of a large video corpus, with methods from conversation analysis and interactional linguists (Stukenbrock, 2009, 2015, 2016). The usability of the model was documented by developmental studies on infants’ capacities to establish reference (Heller and Rohlfing, 2017; Heller, 2019). For the present study, it provides the framework for an investigation of gaze practices used by participants in the course of demonstrative reference.

The jobs (1–10) are accomplished by the participants in temporally flexible ways. If, for instance, participants already maintain “an eye-to-eye ecological huddle” (Goffman, 1963, p. 95), they will not have to establish focused interaction in order to jointly attend to an object. In contrast, speakers will have to mobilize additional resources to summon disattending addressees with whom they are currently not engaged. The first job thus defines the interactional precondition for attention sharing. The subsequent jobs detail the specific tasks that participants are faced with according to their role as referring participant (P) and addressee (A).

**(1) Establishing Focused Interaction**

In order to jointly attend to a visible phenomenon in their surroundings, participants may have to establish *focused interaction* first (Goffman, 1963). In contrast to *unfocused interaction*, in which persons are merely together in the same situation and may glean information about one another without getting engaged, *focused interaction* “occurs when persons gather close together and openly cooperate to sustain a single focus of attention, typically by taking turns at talking” (Goffman, 1963, p. 24). When persons are momentarily not in focused interaction but within reach of one another, they sometimes use demonstratives to (re-)engage another person in focused interaction.

**(2) Projecting a Domain of Pointing**

Participants who are in focused interaction may use demonstratives to share attention to visible objects with their addressees. In order to direct A’s visual attention to an object, P first has to orient him- or herself in space. Prior to initiating joint attention, P can thus be seen to look at the object before sharing it with A. In other words, P projects a *domain of pointing*.

Depending on the local context, P’s self-orienting practices may be witnessed by A. We can therefore expect that P’s self-orientation may be used by A to anticipate a relevant domain by following P’s line of regard. This meta-perceptive gaze practice of observing the visual orientation of the partner and following his or her gaze to a new domain will be investigated in the section on “Deictic Reference, Speaker Gaze Following, and Joint Attention.”

**(3) Establishing the Perceptual Relevance of the Body as a Semiotic Resource**

In order to direct A’s visual attention to an object, P has to make sure that his or her gesture is visible to A. P has to establish the perceptual relevance of his or her own *body as a semiotic resource* to be attended to by A. This can be done through verbal summons, salient body movements, touch, etc. and, most importantly, verbal deictics. As has been argued, verbal deictics constitute a central gaze-summoning device in face-to-face interaction (Streeck, 2002; Stukenbrock, 2009, 2011, 2018a,c).

We can expect P to monitor the success of his or her attempt to secure addressee gaze (Goodwin, 1980, 1981). Gaze shifts from P to A may not only occur at the end of P’s utterance. Instead, we can expect A-gaze monitoring by P while his or her referential action is still emerging. This meta-perceptive practice of monitoring the addressee’s gaze will be investigated in the section on “Deictic Reference, Addressee Gaze Monitoring, and Joint Attention.” In the model, it is defined as job 9 (below).

**(4) Demonstratives and (5) Pointing Gestures as Multimodal *Gestalts***

Gesturally used deictics need a pointing gesture to direct A’s visual attention to the target. The unity of verbal and gestural components in demonstrative reference has been conceptualized as a multimodal *Gestalt* (Heath, 1985; Goodwin, 2003; Streeck, 2009; Stukenbrock, 2011, 2015; Mondada, 2015). Note that, while the demonstrative has to be heard and understood, the concurrent gesture has to be seen by the addressee. In order to maximize the opportunity for successful reference, demonstratives and gestures are deployed in context-sensitive, temporally flexible, and recipient-designed ways (Hindmarsh and Heath, 2000). Their flexible use serves to synchronize the performance of P’s gesture with A’s gaze allocation to P (Stukenbrock, 2018c). Their multimodal packaging and local timing crucially depends on A’s activities. Again, monitoring the addressee (job 9) helps P to maximize interpersonal coordination with A (cf. section “Deictic Reference, Addressee Gaze Monitoring, and Joint Attention”).

**(6) Constituting a Domain of Scrutiny**

In general, it is assumed that A uses P’s pointing gesture to extrapolate a linear vector to the target (Fillmore, 1982, p.46). However, in naturalistic settings with dense perceptual and cognitive ecologies, locating the target is a complex task (Stukenbrock, 2009, p. 305f.); it cannot be reduced to geometrical operations as in controlled experimental settings (Butterworth, 2003). Instead of extrapolating a vector, A first has to constitute a *domain of scrutiny* (Goodwin, 2003, p. 221) within which the *target* is to be found.

**(7) Identifying the Target**

After the domain of scrutiny has been established, A has to identify the *target*. Depending on contextual factors such as distance, complexity, accessibility, and transparency of the domain of scrutiny (Goodwin, 1996), identifying the target may either be unproblematic or lead to repair sequences. The perceptual task of identifying the target is intimately connected to the cognitive task of constructing the referent. Monitoring A while he or she is fulfilling this task may help P to anticipate success, or failure, of the referential act.

**(8) Constructing the Referent**

Standard accounts do not distinguish between target and referent. Following Quine (1960) and Clark et al. (1983), I distinguish between the perceptual task of establishing the target and the cognitive task of identifying the referent (Stukenbrock, 2009, p. 307–309). Although these tasks are normally accomplished as one, the distinction is licensed by repair sequences. These document categorically different problems (repairables) leading to problem-specific repair (Stukenbrock, 2015, p. 302–313). The distinction between target and referent accounts for locally emerging problems that participants themselves orient to as distinct trouble sources.

According to the literature on embodied reference (Hindmarsh and Heath, 2000, p. 1872f.), repair occurs after misunderstandings have been revealed in A’s subsequent turn^2^. However, repair may also occur earlier in the course of a referential action when P looks back at A, anticipates problems, and repairs by dynamically enhancing target visibility (job 7) or facilitating referent construction (job 8). We can expect that addressee gaze monitoring provides a critical resource for participants to foresee trouble and repair early and thus to assure intersubjectivity and progressivity (Heritage, 2007). The following paragraph explains this job (9) in general terms.

**(9) Monitoring Addressee Gaze**

For demonstrative reference to be successful, P has to make sure that it is comprehensible and efficient, i.e., that A perceives what P wants A to perceive (i.e., P’s body as a semiotic resource, domain of scrutiny, target). Monitoring A in the course of demonstrative reference is a powerful instrument for P to shape his or her action in recipient-designed ways. When P gazes at A to monitor A’s gaze orientation, A’s perception becomes the object of P’s perception. Perceiving A’s perception (*Wahrnehmungswahrnehmung*; cf. Luhmann, 1984, p. 560; Hausendorf, 2003) at key moments in the interaction allows P to adapt ongoingly to what P perceives and infers about A’s perception. Addressee gaze monitoring will be investigated in the section on “Deictic Reference, Addressee Gaze Monitoring, and Joint Attention.”

**(10) Display of Understanding**

The referential sequence is brought to a close when A displays understanding in the subsequent turn. A’s display of understanding implies a successful identification of target (7) and referent (8), as well as an unproblematic completion of the preceding tasks. However, A’s response may also document trouble and initiate repair. As has been argued above (jobs 8 and 9), response turns are not the only position in which problems may surface and lead to repair. This may also happen earlier in the sequence.

### Interim Summary

In this section, a model was introduced that explains the interactional organization of deictic reference in terms of the jobs participants have to fulfill in order to establish joint attention on the referent. While formulated on an abstract level, the model is grounded in detailed observations on participants’ actions in face-to-face interaction (Stukenbrock, 2015). The jobs make particular gaze patterns expectable: first, *gaze shifts to objects*, and second, *gaze shifts to coparticipants*. When P sees an object and wants to share it with A, he or she will have to shift gaze between the two, and when A is summoned by P to attend to that object, A will likewise shift gaze between P and the object. According to the model, the two types of gaze patterns, gaze shifts to objects vs. gaze shifts to coparticipants, perform different functions according to their sequential position and the participant role of the gazer (P or A). Notably, P-gaze to an object of joint attention projects a future domain of pointing and serves as self-orientation (job 2) before P initiates joint attention. In contrast, A gaze to the domain of scrutiny (job 6) aims at identifying target and referent. This is accomplished by A on the grounds of his or her perception of P (jobs 3 and 4) and inferences about P’s communicative intentions.

Furthermore, the model states that gaze shifts to coparticipants serve different, context-specific functions. P-gaze to A, and A-gaze to P, when aimed at the other’s eyes, implement meta-perceptive functions. The partner’s perception is turned into the object of perception. According to the model, the interactional function of meta-perceptive gaze practices differs with respect to participant role, sequential context, and temporal implementation. The subsequent analysis is based on the expectation that P-gaze to A, before, during, and after a deictic referencing act serves different functions than A-gaze to P in the course of that act.

## Materials and Methods

### Participants

The study is based on two corpora of video data recorded with mobile eye tracking glasses worn by participants in naturally occurring interaction. The participants were friends or colleagues who engaged with each other as part of their lives. This naturalistic approach to data collection is derived from the endeavor of conversation analysis to reconstruct the “endogenous organization of social activities in their ordinary settings” (Mondada, 2013, p. 33). Two different settings were selected from the range of activities represented in the corpus: shopping together at a market (three dyads), and visiting a museum together (three dyads). The choice was based on the observation that, in both settings, mobile and static configurations emerge naturally as participants collaboratively move on or stop to share attention on objects they consider noticeable in the local context.

### Data Collection and Analytic Procedure

For the first corpus, SMI glasses (sampling rate, 30 Hz) were used. The data for the second corpus (SNSF project DeJA-VI) were recorded with Tobii Pro Glasses II (sampling rate, 50 Hz) and an external video camera to offer a full shot on the activities. The eye tracking recordings and the video from the external camera were frame-precisely synchronized with *Adobe Premiere Pro* and exported as a single split-screen video. The split-screen video and the corresponding wave file were imported into ELAN (Wittenburg et al., 2006) in order to make verbal transcriptions and multimodal annotations. Each split-screen video lasts between 30 and 45 min. Altogether, the verbal transcripts cover roughly 4 h. Talk was transcribed according to GAT2 (Selting et al., 2009; see Supplementary Material). The videos were precoded for all occurrences of gesturally used deictics and concurrent gestures. Gesturally used personal, temporal, and modal deictics were excluded from this study. The detailed analyses drew on a subset of demonstrative reference in which joint visual attention was (1) attested in the eye tracking data (gaze fixations on the target), (2) displayed by the participants in social interaction, and therefore (3) mutually known to the participants (Clark, 1996), as well as (4) accessible to sequential analysis. Twenty sequences were analyzed using methods from multimodal conversation analysis (Schegloff, 2007; Sidnell and Stivers, 2013) and interactional linguistics (Selting and Couper-Kuhlen, 2001). The multimodal annotation was adapted from Mondada (2019; see Supplementary Material). The extracts presented in the subsequent section cover both corpora and exemplify context-specific variations in the use of meta-perceptive gaze practices along with demonstratives and embodied conduct in the course of demonstrative reference.

## Analysis: Meta-Perceptive Practices and Joint Attention in Deictic Reference

Gaze shifts to objects and gaze shifts to coparticipants are part of the sequential orderliness of demonstrative reference and joint attention in ordinary interaction. Both types of gaze shifts may pass unnoticed, or they may be perceived and oriented to by the participants. In this section, I investigate gaze practices that participants use in order to gain access to the coparticipant’s visual perception. As members’ practices, these meta-perceptual practices constitute procedural solutions to the problem of coordinating bodies and minds for demonstrative reference and joint attention. The deployment of meta-perceptive gaze practices is sensitive to spatial, temporal, and interactional affordances and constraints of the context. The aim of the analysis is to uncover how participants’ mutual access to one another’s gaze helps them shape their actions moment by moment according to the action of the other. The analysis starts with speaker gaze following. With respect to the sequential jobs described in the model above, gaze following occurs early and thus shapes the way in which subsequent jobs are accomplished. Next, addressee gaze monitoring will be investigated. It is closely coupled with the speaker’s production of deictics and thus occurs later than gaze following within the sequential ordering of jobs. Last, I will present instances in which meta-perceptive gaze practices are absent, and propose that in the local context, participants’ fine-tuned interpersonal coordination allows them to establish joint attention in implicit ways.

### Deictic Reference, Speaker Gaze Following, and Joint Attention

This section is concerned with the following problems: How is gaze following organized in everyday activities? When does it occur, how does it contribute to demonstrative reference and joint attention, and how is it integrated in the sequential order of social actions? To answer these questions, gaze following is conceptualized as an interactional gaze practice of tapping into a coparticipant’s gaze and exploiting it as a resource to gather information on *where*, *how*, and *for how* long he or she is looking, and to infer *what* he or she is looking at, and *wh*y.

The first extract (“Hokkaido”) is from the market corpus. The figures in the transcript were extracted from the split-screen video and represent the participants’ respective eye tracking recordings. In the figures, P’s perspective is displayed on the left (green gaze cursor) and that of A on the right (blue gaze cursor). The color coding in the transcripts corresponds to the participants’ gaze cursor. The following abbreviations are used: P: referring participant; A: addressee; GF: gaze following; PG: pointing gesture; vb: verbal; gz: gaze; and ge: gesture (see Supplementary Material for details).

The extract exemplifies the projective force of self-orientation by P to a domain of pointing (job 2) and the interactional significance of early gaze following by A for reference and joint attention. It starts after two friends, P and A, have arrived at a local farmers’ market. They have talked about different sorts of pumpkins for sale at the first stall. A’s preference for Hokkaido constitutes the common ground for P’s subsequent noticing. The noticing refers to a sign indicating Hokkaido. It contains a demonstrative and is accompanied by a pointing gesture (l. 3). However, the resource used by A to co-orient with P is not his gesture but his gaze. An analysis of the temporal details demonstrates that A taps into P’s gaze orientation and follows his line of regard early. Consequently, her gaze is already in place when the reference occurs.

At the beginning of the extract (l. 1), the participants are in an open state of talk (Goffman, 1981, p. 74, 134). P is looking at a sign indicating Hokkaido (Figure 1, left) when A turns toward him (Figure 1, right) and begins to follow his line of regard. Focused interaction (job 1) is established when they simultaneously start talking (l. 2 and 3). While A’s utterance (l. 2: sollen wir/“shall we”) projects a proposal and is broken off in the course of the overlap, P’s turn is continued (l. 3). It contains a spatial deictic coupled with a gesture and followed by a nominal phrase (l. 3: dA steht AUCH hokkaido dran;/“there it also says hokkaido”).

**EXTRACT 1:**
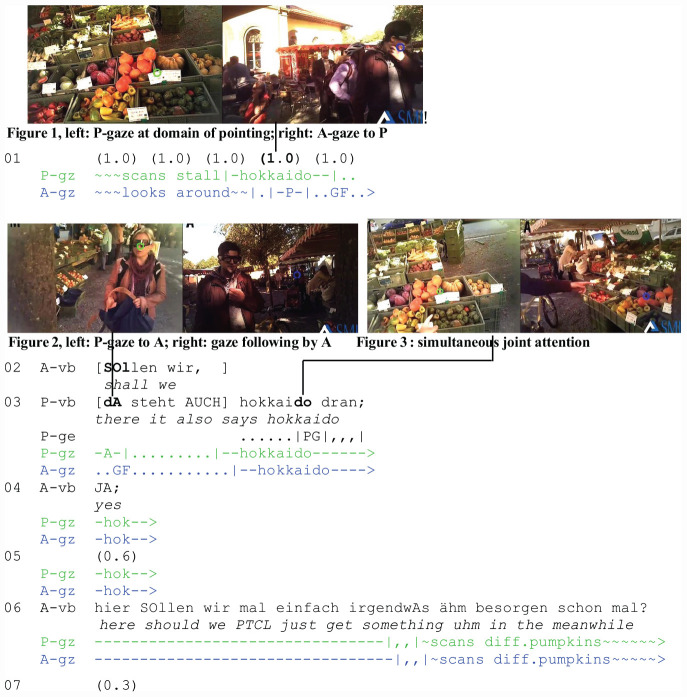
“Hokkaido” (WM02_00:13:20)

At the beginning of his utterance, P shifts his gaze to A (Figure 2, left). He can see that she is moving her gaze toward the domain he has been looking at before (Figures 2, 3, right). When P shifts his gaze back to the pumpkins (Figure 3, left), joint attention has been established (Figure 3, left and right). A’s confirmation (l. 4: JA/“yes”) reveals successful, unproblematic reference. The participants’ focus of attention is sustained as they synchronously scan the objects for a while and A ventures a buying proposal (l. 6).

Due to the temporality of its occurrence, gaze following plays a privileged role among the practices that contribute to the moment of joint attention. A taps into P’s line of regard early and follows it to the phenomenon that P has been looking at before he refers to it. At this point, A is perceptually already orienting to the domain of scrutiny (job 6). Thus, P’s referential act (jobs 4 and 5) helps her identify target and referent, but does not initiate her gaze shift to the target.

The second extract (“Balcony”) is from the market corpus as well. The participants return from shopping together and arrive at the researcher’s house. A is talking about her party when P interrupts her to comment on the facade of the house. In contrast to the first extract, the participants are already in focused interaction (job 1). The example demonstrates the occurrence of gaze following in an ongoing conversation. Whereas in extract 1, gaze following was initiated by A before the participants started talking, it is now set off by P’s interruptions and occurs concurrently with talk. In the figures in the second transcript, P’s perspective is situated on the left (green cursor) and that of A on the right (blue cursor).

A has complained about a friend who cannot come to her party. She closes the topic with an assessment (l. 13–14), and continues planning (l. 16) when she is interrupted by P who utters a noticing (l. 17). In the course of the interruption, A looks at P’s eyes (Figure 4, right) who is, however, not reciprocating her gaze but looking upwards (Figure 4, left). A continues her turn and projects an account (l. 19), but P interrupts her again by proposing an improvement related to the house (l. 20).

**EXTRACT 2:**
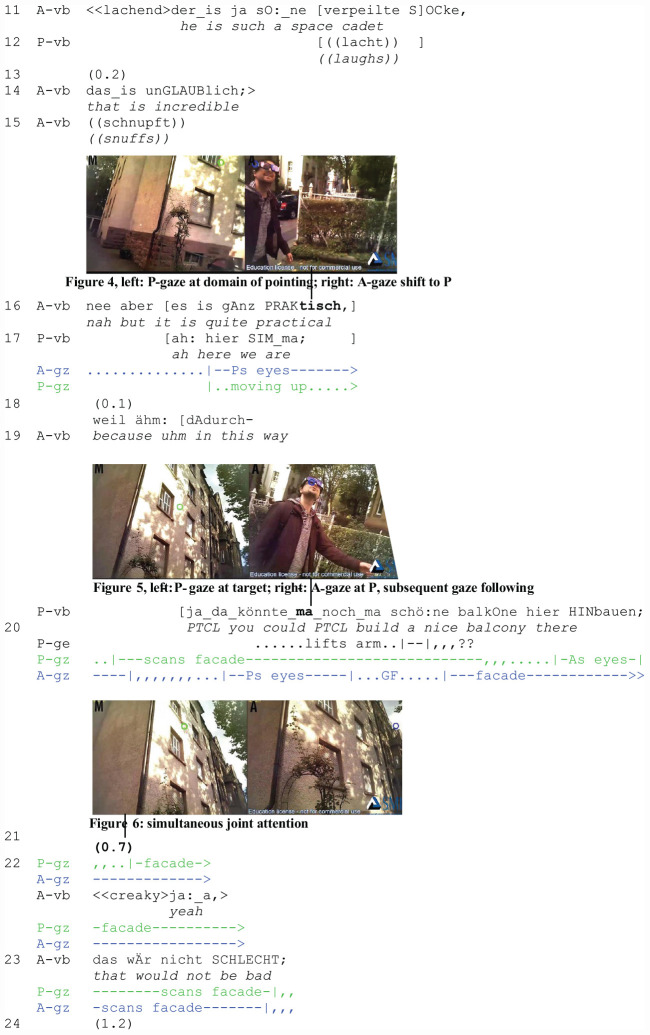
“Balcony” (WM02_00:34:41)

Upon the second interruption (l. 20), A shifts her gaze once more to P’s eyes (Figure 5, right). She can now see that he is still scanning the facade (Figure 5, left). A follows his line of regard (job 6) and also looks at the facade (l. 20–23) (Figure 6, right). After a pause (l. 21), she utters a response token (l. 22: ja:_a/“yeah”), which displays her understanding (job 10). It implies a successful identification of target (job 7) and referent (job 8). A’s subsequent assessment (l. 23) aligns with P’s stance toward the house. Thus, joint attention (Figure 6, left and right) as a mutually known visual, cognitive, and affective orientation to an object has been accomplished.

Several practices contribute to joint visual attention and intersubjectivity: P’s noticing (l. 17) and his subsequent assessment (l. 20) are both sequentially placed as interruptions and solicit A’s gaze (job 3). They entail, however, different gaze patterns. Whereas the first instance of gaze shift to P does not prompt A to follow P’s gaze to the target, the second instance does. In contrast to the noticing (l. 17) that contains a deictic reference to the participants’ present location in space and is interpretable without visual evidence, the subsequent assessment (l. 20) can only be responded to by A on the grounds of visual evidence. It necessitates perceiving what the speaker perceives in order to understand what he refers to.

Therefore, P’s upward gaze acquires a different interactional status in the second instance: it is perceived by A as displaying relevant spatial information (job 3) and used as a directional cue. A taps into P’s gaze and disregards the vague gesture that he performs too low for her to attend to. Due to the unperspicuousness of the gesture and the temporal precedence of gaze following, the former remains interactionally irrelevant. Instead of the gesture, speaker gaze following contributes to the emergence of joint attention in this sequence.

The third extract (“Model”) is from the museum corpus. In the transcript, A’s perspective is displayed on the left (blue cursor) and P’s on the right (green cursor). Two friends are at the Uniseum, the museum of the University of Freiburg/Br. The extract exemplifies an instance of unsuccessful gaze following and contains several repairs. As in extract 1, the participants are not in focused interaction. For various reasons, visual attention sharing is more difficult: the museum visitors are further apart than the participants at the market. They have established divergent foci of visual attention and are not available for mutual engagement. Instead of bodily adjustments, one of them has to walk over to the other to create a new interactional space. We join the participants when P summons A to look at an exhibit in the gynecological section while A is reading an explanation about moulage. The gaze following occurs during the pause in l. 4. The analysis focuses on the repair sequence initiated by A (l. 5) after A has turned to P and tried to follow P’s line of regard.

The participants are several meters apart and have established divergent foci of attention (Figure 7, left and right) when P summons A to share an exhibit with her. It is a model of the expulsion phase of childbirth, as P has learnt from reading the label. When P summons A, she refers to the model with a demonstrative (l. 1: das/“that”).

**EXTRACT 3:**
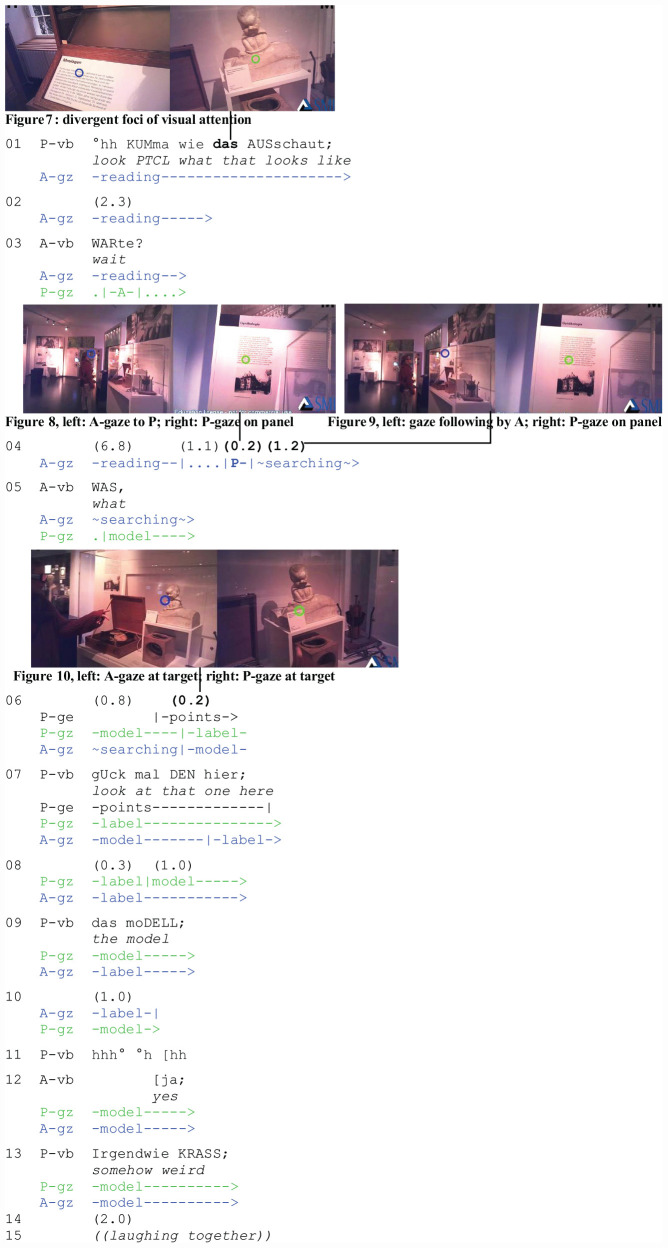
“Model” (Uniseum_01_00:32:26)

Instead of shifting her gaze to P, A asks her to wait (l. 3) and finishes reading. Subsequently, she turns round, walks to P (Figure 8, left), and follows P’s line of regard (Figure 9, left) without, however, being able to narrow down the domain of scrutiny (job 6) and to identify the target (job 7). The search is documented in the eye tracking data by scanning eye movements across text panels and exhibits. A initiates repair (l. 5: WAS/“what”): the interrogative marks the demonstrative in P’s summons (l. 1: das/“that”) as the repairable (job 4).

P repairs (l. 7) by referring to the exhibit with a demonstrative (DEN/“that”), a proximal local deictic (hier/“here”), and a pointing gesture (jobs 4 and 5). The gesture precedes speech and directs A’s gaze to the domain of scrutiny and target (jobs 6 and 7) before the utterance begins (Figure 10, left). P’s utterance is designed to help A identify the target referent (jobs 7 and 8) at the moment in which her gaze arrives on site. A, however, does not respond. In contrast to extracts 1 and 2 in which displays of understanding (job 10) indicated successful reference, A’s silence (l. 8) in extract 3 constitutes a noticeable absence.

The pause that ensues is taken by P as an indication that the problem persists. This is documented in P’s subsequent turn: P repairs the deictic reference in her previous turn (l. 7: DEN hier/“that one here”) with a coreferential nominal phrase (l. 9: das moDELL/“the model”). Sequentially, a response is expected, which displays A’s understanding (job 10) and brings the repair sequence to a close. However, A still remains silent (l. 8). Why does she still withhold a response?

Note that in order to close the sequence as a whole, it does not suffice to display successful reference. From an interactional perspective, more is at stake. A also has to respond to P’s implicit invitation to assess the referent^3^. The referential problem is thus inextricably linked to the problem of an expectable but missing assessment that A may find difficult to offer. Assessments are acts of participation in social activities (Pomerantz, 1984). They require and claim knowledge of the assessed referent. Refusing to assess a referent may index insufficient knowledge or trouble with the referent.

After another pause (l. 10) and an intake of breath that projects further talk by P (l. 11), A finally responds with a minimal acknowledgment token (l. 12: ja/“yes”). However, she does not engage with the exhibit any further. While the problem of localizing and identifying the referent has now been resolved, the interactional problem of assessing the exhibit persists. A’s refusal to assess it is even more evident now. P orients to this and no longer pursues an assessment from A. Instead, P now offers an assessment herself (l. 13: Irgendwie KRASS/“somehow weird”). A, however, withholds a second assessment (Pomerantz, 1984). The sequence comes to a close as the participants, after another pause (l. 14), laugh their embarrassment at the gynecological model off (l. 15), and move on.

The extract shows that demonstrative reference is part and parcel of social actions such as assessments. Understanding the function of demonstratives, pointing, and gaze following in everyday activities is thus inexorably linked to understanding how their actual use is shaped by and continuously adapted to the unfolding interactional context.

To sum up, extracts 1 and 2 have exemplified instances in which speaker gaze following constitutes an important practice of establishing joint attention. Significantly, gaze following occurred early, i.e., the gaze follower, A, anticipated the domain of scrutiny (job 6) before it was indexed by demonstratives and pointing gestures in P’s utterance (jobs 4 and 5). In both instances, verbal responses (job 10) documented successful reference by A in second position, i.e., after turn-taking. Extract 3, in contrast, exhibited an extended repair sequence. After a request to wait and an unsuccessful attempt at gaze following, A initiated repair (l. 5) with an interrogative (l. 5: *was*/“what”). Beyond verbal repair markers, delays or silences may also indicate referential problems. However, non-responding addressees may be silent for reasons other than unsuccessful reference. In extract 3, A’s silence implemented her refusal to assess the referent. This led to a further repair by P until the two components of the expected response, acknowledging the referent and delivering an assessment, were untied. Whereas the referent was finally acknowledged by A, she never delivered an assessment. Extract 3 differs from extracts 1 and 2 in another respect: Not only is speaker gaze following unsuccessful, addressee gaze monitoring (see next section) is also absent. After briefly shifting her gaze to A at the very beginning, P never shifted her gaze to A again subsequently. The embarrassment at the gynecological model that prevented A from responding also forestalled mutual gaze between the participants, and it prevented P from addressee monitoring throughout the sequence. Consequently, P lacked important cues regarding A’s participation and the (non)emergence of joint attention.

### Deictic Reference, Addressee Gaze Monitoring, and Joint Attention

Verbal responses display addressees’ understanding (job 10) of speakers’ prior actions after turn-taking has occurred. In demonstrative reference, A’s gaze orientation constitutes early evidence for P whether joint visual attention is emerging. This section investigates addressee gaze monitoring as a meta-perceptive practice of P to maintain intersubjectivity continuously in the course of reference. This minimizes the occurrence of extended repair sequences such as in extract 3.

We return to the balcony sequence (Extract 4: “Balcony Revisited”) and analyze the temporal placement of P’s gaze shift to A with respect to the sequential ordering of the participants’ jobs. The example demonstrates that by shifting gaze from the target to A while P’s initiating action is still underway, P gains evidence on how A is responding before the initiating action is closed. We join the participants when P interrupts A for the second time (l. 20) and focus on P’s gaze shifts (green cursor) between the facade and A (blue cursor).

In the course of his utterance (l. 20), P continues to scan the facade. Toward the end, he shifts his gaze to A (Figure 11, left) who has stopped talking (l. 19). By looking at her eyes (Figure 11, left), P can see and infer that A is now looking at the facade as well (Figure 11, right). A moment of perceiving A’s perception occurs (job 9). Shifting gaze to A and checking her visual orientation is an observable gaze practice in deictic reference (Stukenbrock, 2009, 2015, 2018c). In this extract, it occurs at a specific position within the sequential order of jobs, i.e., immediately after P’s referential act and before A’s response. Addressee gaze monitoring is used by P in a position-sensitive way, it is temporally coordinated with the jobs that A is expected to fulfill next. As will be shown below (extracts 5 and 6), this is not the only position for addressee gaze monitoring in deictic reference.

**EXTRACT 4:**
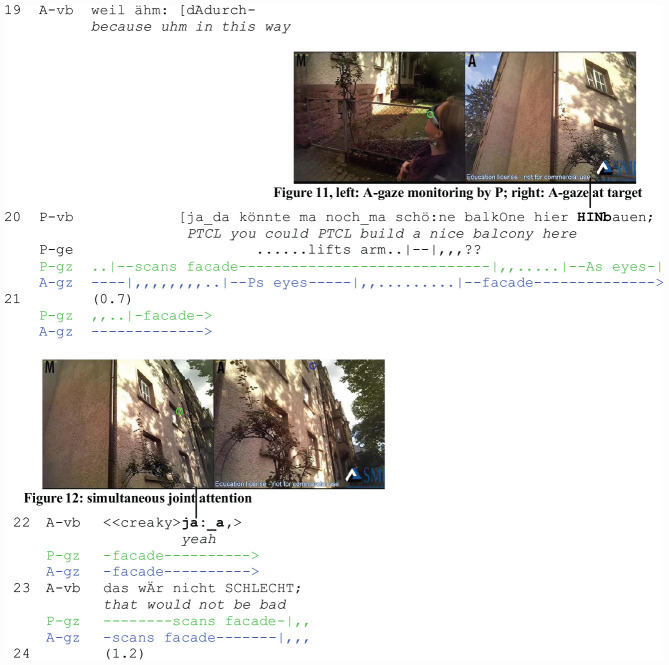
“Balcony Revised”

During the pause in l. 21, P shifts his gaze back to the facade. When A utters a response token (l. 22: ja:_a/“yes”) and aligns with P’s assessment (l. 23: das wÄr nicht SCHLECHT/“that would not be bad”), both are looking at the facade at the same time (Figure 12, left and right). A phase of simultaneous joint attention is thus established, one that is verbally confirmed (job 10) and thus mutually known by the participants. The temporal ordering reveals that A-gaze monitoring before the end of the utterance allows P to gather information about A’s compliance and visual co-orientation before turn-taking occurs and a verbal response (job 10) is due. A-gaze monitoring is a practice of assessing online the outcome of initiating joint attention.

In the next example (Extract 5: “Carnival Game”), P engages in addressee gaze monitoring twice, before (l. 1, Figure 13) and after (l. 3, Figure 14) demonstrative reference (l. 2). In the transcript, A’s perspective is on the left (green cursor) and P’s perspective on the right (red cursor). The data are from recordings made in the Swiss Museum of Games. The participants, two friends who speak Swiss German, take a tour through the museum. In contrast to extract 4, addressee gaze monitoring is facilitated by the spatial configuration of the museum visitors. In contrast to the friends who return from the market and are walking while talking, the museum visitors have stopped in front of a showcase with games. They have established an interactional space that gives them access to the exhibits and to each other’s faces. The following analysis highlights the temporal and contextual sensitivity of A-gaze monitoring and its function in establishing joint attention.

**EXTRACT 5:**
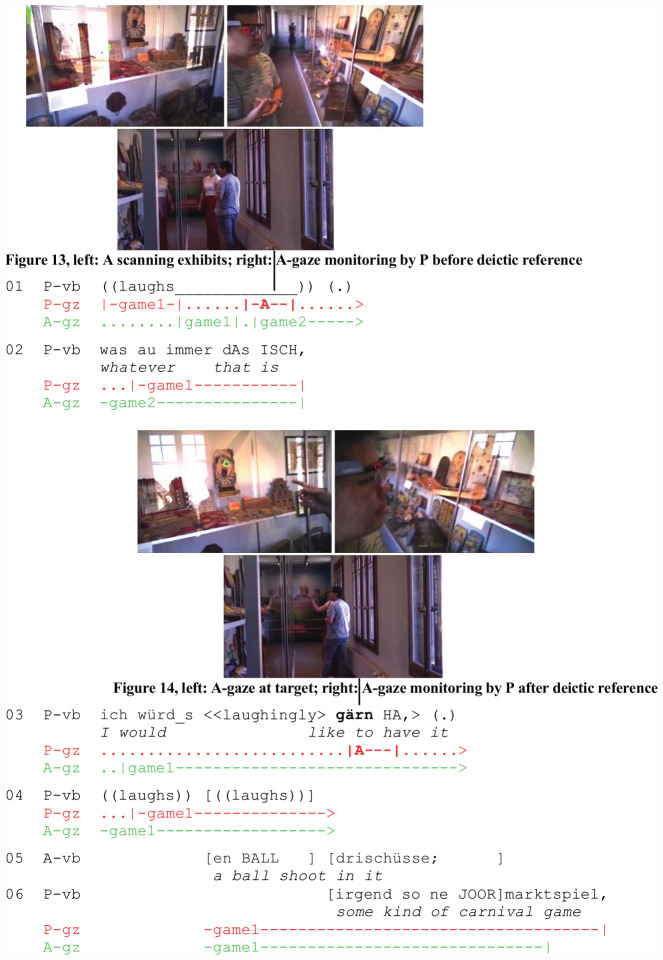
“Carnival Game” (SM02_00:03:32)

Amidst the games in the showcase, P notices an unidentifiable exhibit, displays her surprise by laughter (l. 1), and in the course of laughing shifts her gaze to A. When P gazes at A (Figure 13, right), A is looking at a different exhibit (Figure 13, left). Subsequently, P formulates her stance toward the target exhibit (l. 2–3), referring to it with a demonstrative and a pointing gesture (jobs 4 and 5). At the end of P’s deictic act, A shifts his gaze to the target (jobs 6 and 7) and keeps looking at it through the second half of P’s turn (l. 3). When P shifts her gaze to A toward the end of her utterance (job 9), she can infer from his gaze orientation (Figure 14, right) that he is now attending to the same exhibit (Figure 14, left). Joint attention and successful reference (job 8) are further displayed (job 10) in A’s comment (l. 5), which overlaps with P’s laughter (l. 4) and talk (l. 6).

The sequential positioning of A-gaze monitoring differs from that in extract 4 in contextually shaped ways. The first A-gaze monitoring occurs concurrently with P’s laughter (l. 1) and serves to check A’s attention and availability. In the sequential context, laughter functions as an attention-getting device designed to engage the coparticipant. It also functions as a preinvitation to A to share the object of P’s laughter. In order to do so, A has to identify the reason for P’s laughter and orient to it. P’s subsequent turn is designed to engage A further. The demonstrative reference is part and parcel of a riddle (l. 2: was auch immer dAs ISCH/“whatever that is”), which invites coparticipation in solving it. In the second part of her turn (l. 3), P playfully expresses a desire for the exhibit and monitors A’s gaze for the second time.

To sum up, P constructs a three-step sequence designed to engage A in sharing the exhibit with her:

(1) P-laughter as an attention-getting device and preinvitation, first A-gaze monitoring to check A’s interactional and perceptual availability (l. 1),
(2) Demonstrative reference as part of a riddle (l. 2),
(3) Playful expression of stance toward referent, second A-gaze monitoring (l. 3).

By perceiving A’s perception (job 9) at this particular moment, P can infer that A has oriented his visual attention to the object. Subsequently, A’s verbal response displays successful reference (job 10). He offers a candidate solution to the riddle by replacing the demonstrative in P’s utterance with a lexical item marked by hedges (l. 6: irgend so ne JOORmarktspiel/“some kind of carnival game”).

This section concludes with an analysis of A-gaze monitoring in a triadic encounter (Extract 6: “Gambling Table”). Two colleagues and friends, C and T, are at the Swiss Museum of Games. Before they visit the exposition, they meet with the head of the museum (A) to plan a conference at the museum’s event hall. We join the participants when they change topic from organizational (l. 11–17) to spatial arrangements (l. 18–24). The deictic reference occurs in a question asked by C and addressed at A, the head of the museum (l. 18-21). C is the pointing participant. In the figures in transcript 6, C’s perspective is displayed on the right (red cursor) and T’s perspective on the left (green cursor). The head of the museum is not wearing eye trackers. The bottom of the split screen displays the recording of the external camera.

After organizational matters have been settled, the participants close the topic (l. 11–15). C and T engage in mutual gaze (l. 11), C then withdraws her gaze from T, vaguely orients to A and the surrounding space (l. 12). When she utters the final closing device (l. 15: voiLÀ/“right”), she has already shifted her gaze to the surroundings (Figure 15, right), projecting a future domain of pointing (job 2). In contrast, her colleague T is still looking at the head of the museum (Figure 15, left).

**EXTRACT 6:**
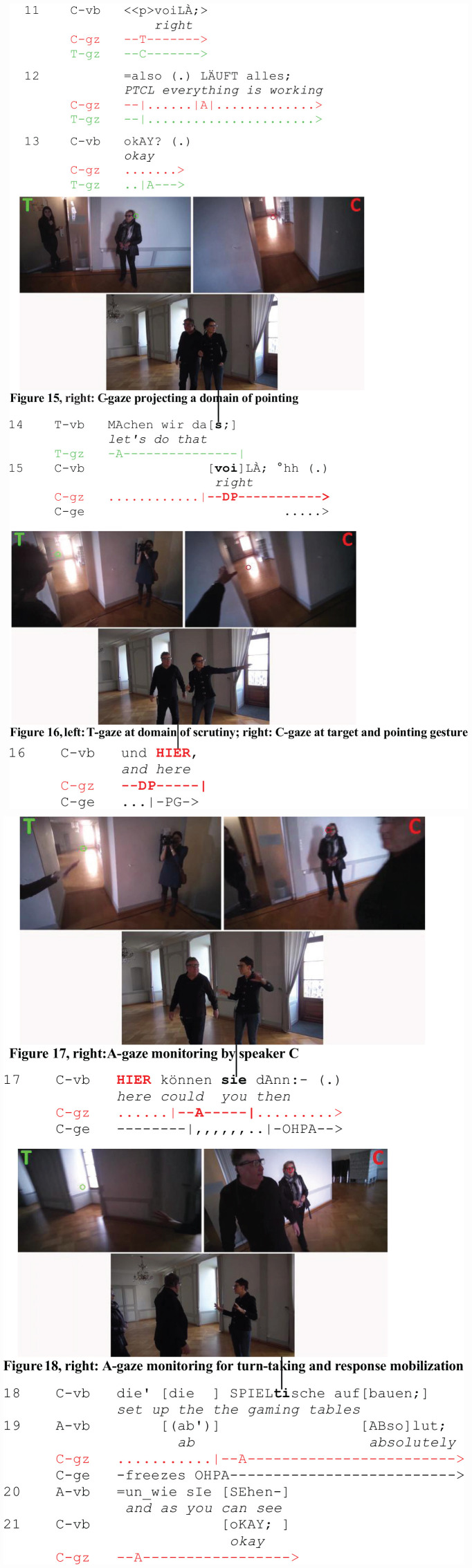
“Gambling Table” (SM01_Saal_00:01:33:12)

C keeps looking at the domain of pointing (job 2) as she utters the first deictic (l. 16: HIER/“here”) and performs a concurrent pointing gesture (jobs 4 and 5). The preparation phase of the gesture precedes the beginning of the turn; its apex is synchronized with the articulation of the first deictic (Figure 16, right). T, who is not directly addressed by the speaker, follows her pointing act and shifts his gaze to the domain of scrutiny (Figure 16, left).

The addressee of the utterance is the head of the museum, A, who is not wearing eye trackers and currently not visible in the video recording. Note that, at this point (l. 16), the participant roles of the addressed and the unaddressed recipient are not yet evident. Only in the course of the next turn-constructional unit (l. 17), when C shifts her gaze back to the head of the museum (A) and selects her with the VOS pronoun (l. 17: SIE/you), is the participation framework of this utterance established and the type of social action (a request) projectable^4^. T, who does not yet know that he will not be the addressee, shifts his gaze to the domain of scrutiny (job 6). This is valid eye tracking evidence (Figure 16, left) for the gaze-summoning function of deictics in demonstrative reference. By gazing at the domain of scrutiny before the VOS pronoun is uttered, he follows C’s referential act continuously and prepares for the role of potential next speaker.

With less technological precision, the head of the museum (A) can be observed looking at the domain of scrutiny as well when speaker C shifts her gaze to A (Figure 17, right). A-gaze monitoring (job 9) allows C to perceive the perception of A and check online whether the referential act be successful.

Significantly, C’s first gaze shift to A (job 9) is temporally placed within the uncompleted utterance, after the second occurrence of the proximal deictic HIER/“here” (l. 17). In both instances, the deictic bears the focal accent and is accompanied by a gesture (Figure 16). In the course of the second part of her utterance, C withdraws her gaze from A (l. 17–18). Toward the end of her utterance, C shifts her gaze back to A (Figure 18, right) who makes a broken-off attempt to respond (l. 19: ab’) and then confirms C’s request (l. 19: ABsolut/“absolutely”).

C’s gaze shift to A toward the end of the utterance is consistent with findings on the function of gaze in turn-taking (Kendon, 1967; Auer, 2020). Whereas the first, turn-internal gaze shift to A is closely tied to the referential act and serves addressee gaze monitoring (job 9), C’s final gaze shift to A is motivated by the end of her turn. C’s turn implements a request that makes a type-conforming second action relevant (i.e., granting or declining the request). Gaze allocation to A selects her as next speaker and serves to mobilize A’s response (Stivers and Rossano, 2010).

Interim Summary: The previous sections have shown that meta-perceptive gaze practices, i.e., speaker gaze following and addressee gaze monitoring, contribute to the successful establishment of joint attention in deictic reference. Addressees who follow speaker gaze early may anticipate the domain of scrutiny. Their gaze thus arrives on the scene before, or at the moment in which talk further elaborates target and referent (previous section). Likewise, speakers may use different sequential positions to shift gaze between target and addressee to monitor A’s visual attention and adapt to it ongoingly (this section). These practices are deployed in context-sensitive ways; they reflect the context of their use and at the same time contribute to the emergence of that context. Absence of mutual monitoring may create, or sustain, problems that disturb the sequential order, threaten intersubjectivity, and lead to extended repair sequences (extract 3). However, absence of mutual monitoring does not necessarily cause problems, as will be shown in the last section.

### Absence of Mutual Gaze Monitoring and the Establishment of Joint Attention

The endogenous organization of a range of social activities emerges in ways that do not invite mutual gaze monitoring, contextual factors making them either unnecessary or preventing them. The examples in this section illustrate that particular spatial configurations and participation frameworks account for variations and non-occurrence of the gaze practices observed so far.

**FIGURE 19:**
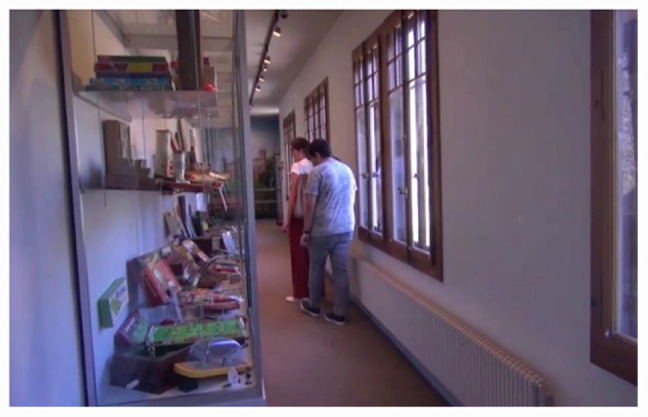
Front-to-back configuration.

**FIGURE 20:**
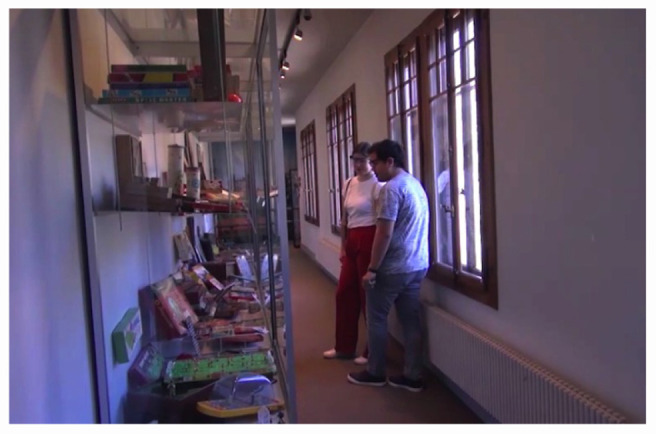
Side-by-side configuration, facing inwards.

The following example (Extract 7: “Magic Robot”) demonstrates a typical case in which contextual factors contribute to the absence of the gaze pattern described in the section on “Deictic Reference, Addressee Gaze Monitoring, and Joint Attention.” Two friends, M and A, are at the beginning of a tour through the Swiss Museum of Games. M is the pointing participant. In the transcript, M’s perspective is displayed on the left (green cursor), A’s perspective is on the right (red cursor). We join them on their way along a corridor with display cases showing a large array of games. They take small steps from case to case, pausing from time to time. The spatial configuration between mobile and stationary phases differs in a slight, but significant way. Whereas the participants’ bodies move into an oblique front-to-back orientation during mobile phases, with A taking the lead (Figure 19), they get into a side-by-side configuration and establish a lateral interactional space (Figure 20) in stationary phases.

The side-by-side configuration, while displaying togetherness, co-orientation, and readiness for attention-sharing, allows them on the one hand to face out of the interactional space centrifugally to establish divergent foci of attention, and, on the other hand, to face inside and arrange themselves “in such a way that their individual transactional segments overlap to create a joint transactional space” (Kendon, 1990, p. 211). The spatial overlap between the participants’ transactional segments, termed “o-space” (Kendon, 1990, p. 211), depends on how far they orient centripetally, i.e., toward the space projected by the coparticipant’s body. The referential act (l. 5) occurs after the participants have stopped and A has started talking (l. 4).

M does not engage in addressee monitoring either before or after the referential act. For the following reasons, it can be dispensed with in the local context: First, the participants are in close side-by-side configuration, slightly turned inwards with overlapping transactional segments. Peripheral vision thus gives them mutual access to one another. Second, focused interaction (job 1) has been re-established verbally by A; M thus knows that A is co-oriented with him. Third, the fact that A engages in displaced talk about her father (l. 4) does not create a visual conflict of interest with the situated noticing initiated by M even though the latter is interruptive. Fourth, the temporal design of the noticing as cutting into A’s talk functions as an attention-getting device. Together with the imperative (l. 5: LUEG/“look”) and the demonstrative (l. 5: DAS/“this”), it serves to underpin the primacy of situated vs. displaced speech and legitimizes the interruption. A aligns and shifts her gaze to the target (jobs 6 and 7) shortly after M’s gesture becomes visible to her (l. 5, Figure 21, right). M and A briefly look at the target simultaneously (Figure 21, left and right)^5^ before M returns to scanning the other exhibits while A continues looking at the target (l. 5–7). When A displays her understanding (job 10) by denominating the referent (job 8) with a proper name (l. 7: magic RObot), M shifts his gaze back to the game, thus creating another moment of joint attention. Visual attention sharing is followed by further talk about the referent. Thus, the establishment of joint attention is mutually known and integrated in the participants’ common ground.

**EXTRACT 7:**
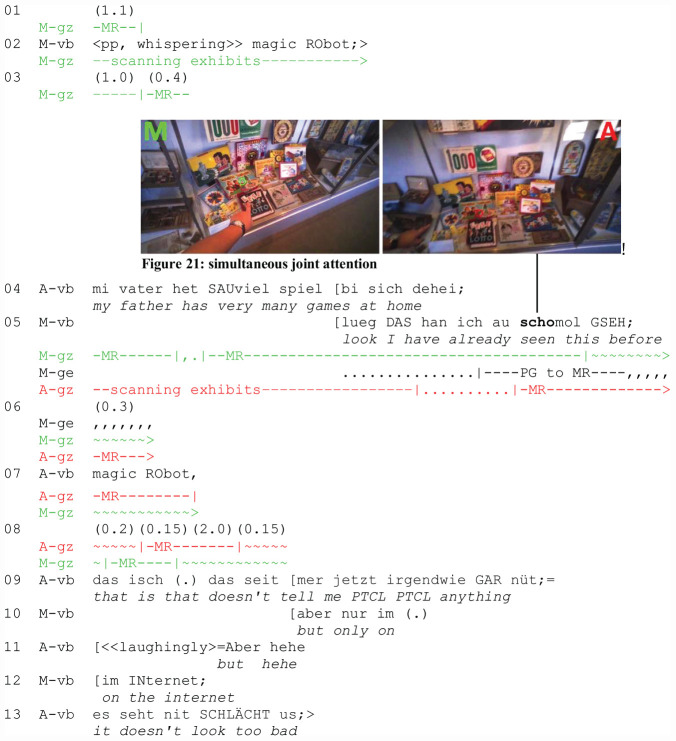
“Magic Robot” (SM02_Rundgang_00:02:30)

None of the meta-perceptive gaze practices described above contributes to visual attention sharing. Instead, a high degree of implicitness is involved. The participants rely on their close side-by-side position, a tacit agreement about visual co-orientation, and – instead of mutual monitoring – on reciprocal inferences about each other’s visual perception. Significantly, part of the inferential work done by A is anchored in overhearing M talking to himself shortly before the verbal exchange starts. While still on the move, M has whispered “magic RObot” to himself (l. 2), indexing an individual discovery he will present as a noticeable to be shared with his coparticipant a few moments later (l. 5). Overhearing a coparticipant’s subdued self-talk may enhance common ground in subtle ways. It resembles acts of unperspicuously observing the other’s visual perception. In more general terms, the overhearing episode exemplifies how participants’ interactional microhistories create intersubjectivity and contribute to the common ground on various levels in explicit and implicit ways.

The last example (Extract 8: “Flipper”) from the Swiss Museum of Games represents another instance in which addressee gaze monitoring is dispensed with. P’s perspective is on the right (red cursor), and A’s perspective is on the left (green cursor). The spatial configuration is the same as in the previous example; it facilitates mutual monitoring and attention sharing by overlapping segments of the visual field. In contrast to the previous extract, the sequential implementation of the referential action is different. The analysis highlights that, beyond particular bodily configurations, the temporal design and type of social action also contribute to a context in which addressee monitoring is dispensed with. In contrast to the attention-getting devices and the type of social action performed in the previous example (interruption, verb of perception in the imperative, noticing), P offers an assessment. Concurrently, P uses a demonstrative and a pointing gesture to direct A’s visual attention to the target game. When A’s gaze arrives at the target (Figure 22, left), P briefly looks elsewhere (Figure 22, right) before shifting her gaze back to the game. An extended phase of simultaneous joint attention ensues (l. 3–5). The referential act is part of an assessment (l. 1: witzig/“funny”) followed by giggling (l. 2). A responds minimally by chuckling (l. 3) and denominating the referent (l. 4: FLIPper), thus displaying successful identification of target and referent. Again, there is no A-gaze monitoring before or after the referential act. Instead, the participants rely implicitly on visual co-orientation and interpersonal coordination; mutual monitoring is dispensed with.

**EXTRACT 8:**
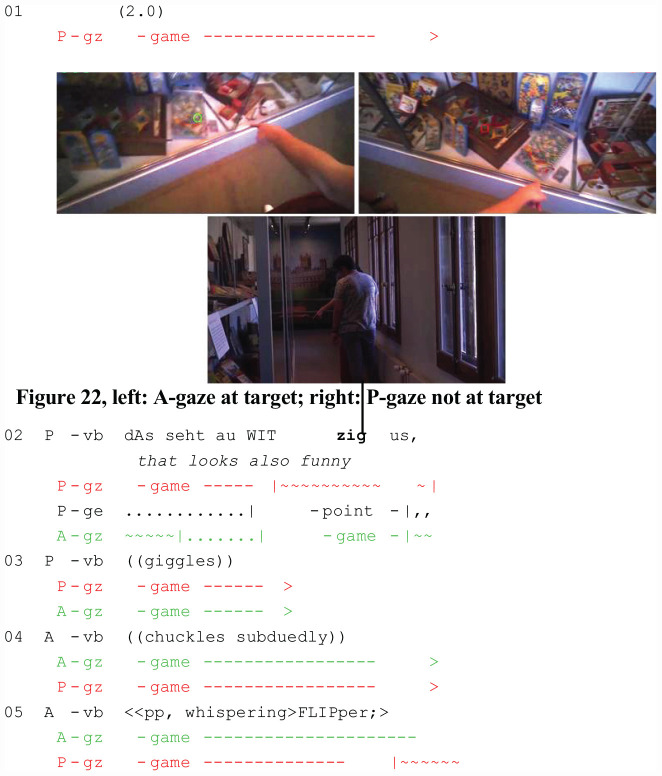
“Flipper” (SM02_Rundgang_00:03:18)

To sum up, the context-related factors that contribute to the endogenous organization of deictic reference and joint attention without mutual gaze monitoring are particular bodily configurations in (semi-)mobile activities. While “an eye-to-eye ecological huddle” (Goffman, 1963, p. 95) is not invited by the activity, participants display to each other bodily and/or verbally that they are together, closely attending to the activity at hand. Thus, individual perceptions can be transformed into shared perception with a minimal array of resources and in sequentially reduced formats.

## Discussion

Based on video data recorded with an external camera and mobile eye tracking glasses worn by participants in naturally occurring social activities, this paper investigated the function of gaze practices deployed concurrently with deictics and embodied pointing to establish joint attention as a mutually known interactional achievement. The focus of the analyses was on meta-perceptive gaze practices that participants themselves oriented to in the course of demonstrative reference, notably, *speaker gaze following* and *addressee gaze monitoring*. The analysis drew on a model of deictic reference which specified the jobs participants have to fulfill sequentially according to their roles as referring participant (P) and addressee (A) in order to establish joint attention and mutual understanding. The jobs were conceived of as participants’ routine solutions to the problem of reference and attention sharing in everyday life.

The methodological challenge of this study consisted of acquiring precise and ecologically valid eye gaze in order to meet with the conversation analytic requirement of preserving the endogenous order of social activities within the context of their occurrence (Mondada, 2013). This was achieved by taking eye tracking technology out of the laboratory to record participants “in the wild.” Mobile eye tracking technology delivered detailed eye gaze data on the participants’ gaze practices without restricting their bodily freedom. Sequential analysis uncovered the participants’ meta-perceptive gaze practices within two activity contexts, shopping at a market and visiting a museum. The analysis revealed that meta-perceptive gaze practices are context dependent, positionally sensitive, tied to the participant roles of P and A, and temporally fine-tuned to the stream of verbal and embodied conduct. It was shown that the temporal placement of these gaze practices with respect to the jobs that participants fulfill in the course of demonstrative reference shape the functions that meta-perceptive gaze acquires in that process.

In the eye tracking data, gaze following appeared as an act of looking at another person’s gaze and following it to a target within a domain of scrutiny. As such, it has three elements: (1) a starting point, the other’s eyes; (2) an end point, the presumed focus of the other’s visual attention; and (3) the trajectory from starting to end point, or from the other’s eyes to the target. This description is in line with conceptualizations of gaze following in developmental studies (Scaife and Bruner, 1975; Flom et al., 2007; Brooks and Meltzoff, 2008, 2014). It lacks, however, an account of the temporality, interactivity, and intersubjectivity of gaze following and joint attention within ordinary activities. The sequential analysis (section “Deictic Reference, Speaker Gaze Following, and Joint Attention”) revealed that, in naturally occurring interaction, gaze following constitutes a complex and heterogeneous phenomenon. It is not initiated by an experimenter who shifts gaze for a predetermined gaze follower to follow. Instead, it emerges online as participants jointly engage with the world, notice things, and notice that their coparticipants notice things. Instead of extrapolating a vector from the other’s gaze, as the metaphor “to follow a person’s the line of regard” suggests, it is a social, interpretive act based on the participants’ mutual assumptions, their involvement in the ongoing activity, and the interactional microcontext. It was argued that the concept of gaze following needs refinement when applied to the investigation of ordinary activities. Gaze following was therefore defined as an interactional gaze practice of tapping into a coparticipant’s gaze and exploiting it as a resource to gather information on *where*, *how*, and for *how long* he or she is looking, and to infer *what* he or she is looking at, and *why*. The inferences that gaze followers drew on their coparticipants’ acts of seeing were socially displayed in how they designed their own next actions and documented understanding of the speaker’s prior turn.

In contrast to speaker gaze following, addressee gaze monitoring does not induce a pointing participant to follow the addressee’s line of regard. Although P may shift gaze from A back to the target (e.g., extracts 1 and 2), or look elsewhere (e.g., extract 5), gaze shifts from A to the target do not constitute instances of gaze following, since P, instead of constituting a new focus of attention, only revisits a target previously established by him- or herself. The sequential analysis (section “Deictic Reference, Addressee Gaze Monitoring, and Joint Attention”) documented that addressee gaze monitoring occurs in the course of, or immediately at the end of demonstrative reference, i.e., before speaker change takes place. It was argued that addressee gaze monitoring is an interactional resource for P to gather real-time evidence on whether joint attention is emerging, and to incrementally add material when they anticipate that intersubjectivity be threatened. Although addressee monitoring is an important means to maintain intersubjectivity in the course of a demonstrative act, it can also be dispensed with (section “Absence of Mutual Gaze Monitoring and Joint Attention”). Mobile settings often complicate visual access to the other’s eyes. In front-to-back and side-by-side configurations, participants often refrain from mutual gaze monitoring. This suggests that the accomplishment of joint attention in mobile, spatially fluid configurations brings about variations in the sequential format of jobs that participants have to accomplish and leads to an absence of gaze practices regularly observable in face-to-face and L-configurations.

This paper has presented qualitative analyses of meta-perceptive practices in two settings. In order to fully understand gaze following and gaze monitoring in naturally occurring interaction, further studies should investigate the occurrence and non-occurrence of these practices in a range of different settings, social activities, and participation frameworks, and take into consideration alternative practices such as eye–hand coordination (Yu and Smith, 2013) which help understand the affordances and constraints that account for participants’ local preferences. Reliable eye tracking data of activities in their natural habitat are needed to build collections of cases that are large enough to uncover the systematicity of context-dependent variations and serve to further develop and refine the interactional model of deictic reference and joint attention. In its current design, the framework offers a high degree of granularity, thus enabling detailed analyses of the interactional jobs that participants need to accomplish in order to establish deictic reference. By accounting for context-dependent variations, it allows for the description of more and less elaborate sequences and formats as well as for a distinction between jobs (e.g., directing the coparticipant’s attention to an object) and resources used to accomplish those jobs (manual pointing, whole body or eye gaze orientation, etc.). In future studies, the usability of the model could be further verified with respect to child development research, and its adaptability to the study of reference and joint attention in atypical interaction, technologically mediated communication, and human robot interaction could be assessed.

## Data Availability Statement

The video and eye tracking recordings for this study are not publicly available because of restricted informed consent of the participants.

## Ethics Statement

Ethical review and approval was not required for the study on human participants in accordance with the local legislation and institutional requirements. The participants provided their written informed consent to participate in this study. Written informed consent was obtained from the individual(s) for the publication of any potentially identifiable images or data included in this article.

## Author Contributions

AS developed the theoretical framework, collected the data with her team, and carried out the empirical analysis.

## Conflict of Interest

The author declares that the research was conducted in the absence of any commercial or financial relationships that could be construed as a potential conflict of interest.

## Footnotes

1 The term “practice” is used in conversation analysis and interactional linguistics to distinguish actions from the practices that are used to implement those actions. It denotes “recurrent ways in which linguistic (and other) resources are used for particular purposes, for instance, in constructing turns, organizing turn taking, initiating repair” (Couper-Kuhlen and Selting, 2018, p. 29).

2 This is, in general, the sequential position for other-initiated repair, i.e., repair launched not by the speaker, but by the addressee, and then left to the speaker to accomplish the actual repair (other-initiated self-repair according to Schegloff et al., 1977).

3 I would like to thank reviewer 2 for pointing out the additional trouble source of an expectable but missing assessment.

4 Concurrently, C transforms her pointing gesture into an open hand palm up shape (OHPU) (Kendon, 2004). A discussion of different gesture shapes is beyond the scope of this paper (see, however, Stukenbrock, 2015, p. 97–230).

5 For reasons of space, the external camera perspective has been cut out in Figure 21.
