# Supplementary material for: Deixis, Meta-Perceptive Gaze Practices, and the Interactional Achievement of Joint Attention
Source: Front Psychol. 2020 Sep 11;11:1779. doi: 10.3389/fpsyg.2020.01779 (PMC7518716; doi:10.3389/fpsyg.2020.01779)
Supplement: Supplementary file 1 [file Table_1.docx]

Supplementary Material

# GAT 2 transcription conventions

(Selting et al., 2009; for the English translation cf. Couper-Kuhlen and Barth-Weingarten, 2011)

Sequential structure

[ ] overlap and simultaneous talk

[ ]

= immediate continuation with a new turn or segment, latching

In- and outbreaths

°h / h° in-/outbreaths of appr. 0.2-0.5 sec. duration

°hh / hh° in-/outbreaths of appr. 0.5-0.8 sec. duration
°hhh / hhh° in-/ outbreaths of appr. 0.8-1.0 sec. duration

Pauses

(.) micro pause, estimated, up to 0.2 sec. duration appr.

(-) short estimated pause of appr. 0.2-0.5 sec. duration

(--) intermediary estimated pause of appr. 0.5-0.8 sec. duration

(---) longer estimated pause of appr. 0.8-1.0 sec. duration

(0.5)/(2.0) measured pause of appr. 0.5 / 2.0 sec. duration

Segmental conventions

and_uh cliticizations within units

uh, uhm, etc. hesitation markers, so-called "filled pauses"

: lengthening, by about 0.2-0.5 sec.

:: lengthening, by about 0.5-0.8 sec.

::: lengthening, by about 0.8-1.0 sec.

Laughter

haha, hehe, hihi syllabic laughter

((laughs)) description of laughter and crying

<<laughing> > laughter particles accompanying speech with indication of scope

Continuers

hm, yes, no, yeah monosyllabic tokens

hm_hm, ye_es, bi-syllabic tokens

ʔhmʔhm with glottal closure, often negating

Accentuation

SYLlable focus accent

sYllable secondary accent

!SYL!lable extra strong accent

Final pitch movements of intonation phrases

? rising to high

, rising to mid

– level

; falling to mid

. falling to low

Loudness and tempo changes, with scope, other conventions

<<f> > forte, loud

<<ff> > fortissimo, very loud

<<p> > piano, soft

<<pp> > pianissimo, very soft

<<all> > allegro, fast

<<len> > lento, slow

<<cresc> > crescendo, increasingly louder

<<dim> > diminuendo, increasingly softer

<<acc> > accelerando, increasingly faster

<<surprised> > interpretive comment with indication of scope

(xxx), (xxx xxx) one or two unintelligible syllables

(may i) assumed wording

# Multimodal transcription conventions

The multimodal transcription of embodied behavior is based on the following conventions (adapted with changes from Mondada, 2019):

P-vb verbal action of participant P

P-gz gaze behavior of participant P

P-ge gesture of participant P

..... action's preparation phase

----- apex and continuation of action

,,,,,, action's retraction phase

~ ~ ~ ~ scanning gaze movements

---> embodied behavior is continued across subsequent lines

| | beginning and end of embodied behavior; if omitted,

the behavior starts or ends before or after the transcript

fig. the exact moment in which the screenshot has been taken, precisely

aligned with the co-occurring speech syllable

# indication of the position in the transcript

# References

Couper-Kuhlen, E., and Barth-Weingarten, D. (2011). A system for transcribing talk-in-interaction: GAT 2 translated and adapted for English. *Gesprächsforschung. Online-Zeitschrift zur verbalen Interaktion* 12, 1–51.

Mondada, L. (2019). Conventions for multimodal transcription. Available at: https://www.lorenzamondada.net/multimodal-transcription [Accessed February 12, 2020].

Selting, M., Auer, P., Barth-Weingarten, D., Bergmann, J., Bergmann, P., Birkner, K., et al. (2009). Gesprächsanalytisches Transkriptionssystem 2 (GAT 2). *Gesprächsforschung – Online-Zeitschrift zur verbalen Interaktion* 10, 353–402.
